# HIV status and participation in HIV surveillance in the era of antiretroviral treatment: a study of linked population-based and clinical data in rural South Africa

**DOI:** 10.1111/j.1365-3156.2012.02928.x

**Published:** 2012-07-30

**Authors:** T Bärnighausen, F Tanser, A Malaza, K Herbst, M -L Newell

**Affiliations:** 1 Africa Centre for Health and Population Studies, University of KwaZulu-NatalMtubatuba, South Africa; 2 Department of Global Health and Population, Harvard School of Public HealthBoston, MA, USA; 3 Centre for Paediatric Epidemiology and Biostatistics, University College of London Institute of Child HealthLondon, UK

**Keywords:** HIV status, HIV knowledge, HIV surveillance, participation, antiretroviral treatment

## Abstract

**Objective:**

To examine whether HIV status affects participation in a population-based longitudinal HIV surveillance in the context of an expanding HIV treatment and care programme in rural South Africa.

**Method:**

We regressed consent to participate in the HIV surveillance during the most recent fieldworker visit on HIV status (based on previous surveillance participation or enrolment in pre-antiretroviral treatment (pre-ART) care or ART in the local HIV treatment and care programme), controlling for sex, age and year of the visit (*N* = 25 940). We then repeated the regression using the same sample but, in one model, stratifying HIV-infected persons into three groups (neither enrolled in pre-ART care nor receiving ART; enrolled in pre-ART care but not receiving ART; receiving ART) and, in another model, additionally stratifying the group enrolled in pre-ART and the group receiving ART into those with CD4 count ≤200/μl (i.e. the ART eligibility threshold at the time) *vs.* those with CD4 count >200/μl.

**Results:**

HIV-infected individuals were significantly less likely to consent to participate in the surveillance than HIV-uninfected individuals [adjusted odds ratio (aOR), 0.74; 95% confidence interval, 0.70–0.79, *P* < 0.001], controlling for other factors. Persons who were receiving ART were less likely to consent to participate (aOR, 0.75, 0.68–0.84, *P* < 0.001) than those who had never sought HIV treatment or care (aOR, 0.82, 0.75–0.89, *P* < 0.001), but more likely to consent than persons enrolled in pre-ART care (aOR 0.62, 0.56–0.69, *P* < 0.001). Those with CD4 count ≤200/μl were significantly less likely to consent to participate than those with CD4 count >200/μl in both the group enrolled in pre-ART and the group receiving ART.

**Conclusion:**

As HIV test results are not made available to participants in the HIV surveillance, our findings agree with the hypothesis that HIV-infected persons are less likely than HIV-uninfected persons to participate in HIV surveillance because they fear the negative consequences of others learning about their HIV infection. Our results further suggest that the increased knowledge of HIV status that accompanies improved ART access can reduce surveillance participation of HIV-infected persons, but that this effect decreases after ART initiation, in particular in successfully treated patients.

## Introduction

HIV surveys and surveillances in sub-Saharan Africa are the main data sources for HIV prevalence and incidence estimates (Boerma *et al.* 2003; Rice *et al.* 2007; Wambura *et al.* 2007; Zaba *et al.* 2007), which are essential indicators for HIV treatment and prevention policy. However, large proportions of eligible persons commonly refuse to participate in HIV surveys and surveillances. For instance, in the nationally representative Demographic and Health Surveys (DHS), the proportions of people refusing to provide a blood sample for HIV testing has ranged from 3 to 33% across countries and years (Hogan D, Salomon JA, Canning D, Hammitt JK, Zaslavksy A & Bärnighausen T, under review). Previous studies have suggested that HIV-infected persons are less likely to consent to participate in HIV surveys and surveillance than HIV-uninfected persons (Reniers & Eaton 2009; Bärnighausen *et al.* 2011). Possible reasons for this relationship include the fear to confirm one’s suspicions of HIV infection and the fear that other people might learn one’s positive HIV status. If HIV status does indeed determine participation, HIV prevalence estimates based on measured HIV status will be biased, and conventional approaches to control for selective participation based on observed factors, such as single and multiple imputation or propensity-score re-weighting, will fail to generate unbiased estimates (Bärnighausen *et al.* 2011).

In this study, we use a novel data opportunity – the linkage of clinical data from an HIV treatment and care programme to data from a large, longitudinal, population-based HIV surveillance in rural South Africa – to investigate the hypothesis that HIV status determines consent to participate in the surveillance. To this end, we examine consent to participate in one of Africa’s largest longitudinal HIV surveillances, conducted by the Africa Centre for Health and Population Studies (Africa Centre) in rural KwaZulu-Natal, South Africa. Like other HIV surveys and surveillances, such as the DHS, the Africa Centre surveillance currently does not make HIV test results available to participants, but instead provides information on location and opening hours of the public-sector HIV testing facilities, where rapid HIV tests can be obtained free of charge. Many of these testing facilities are located within primary health care clinics, on the same premises as antiretroviral treatment centres, ensuring that HIV-infected patients can be offered CD4 counts and ART counselling in immediate proximity to the HIV testing facility.

As the HIV surveillance itself does not provide information on HIV status, fear of confirming a suspicion that one is HIV-infected through participation in the surveillance is not a plausible reason why HIV-infected persons may be more likely to refuse to participate than HIV-uninfected persons. However, fear that others might learn about one’s positive HIV status is a plausible reason, if the persons who are eligible to participate in the surveillance do not trust the fieldworkers’ assurances that HIV test results will be kept confidential. However, in order for this reason to affect HIV consent differentially by HIV status, some proportion of eligible persons must know or suspect their status. One source of information on HIV status knowledge is the HIV treatment and care programme. Patients enrolled either in pre-antiretroviral treatment (pre-ART) care or receiving ART will certainly know their positive HIV status. The effect of HIV status on consent to participate in HIV surveillance is necessarily transmitted through status knowledge. If this effect did indeed exist, we would expect that patients enrolled in pre-ART care or receiving ART would be less likely to consent to participate in the surveillance than HIV-infected people who have not yet sought HIV treatment or care, because it is likely that some proportion of the latter group do not know their status. In this study, we first test this prediction. We then discuss our findings, considering alternative explanations and implications for health policy.

## Methods

### Setting and surveillance

This study took place in a poor rural community in the Hlabisa sub-district of northern KwaZulu-Natal. Adult HIV prevalence in the community is above 20% and peaks at more than 50% in women aged 25–29 years and 44% in men aged 30–34 years (Welz *et al.* 2007). Adult HIV incidence has been consistently found to be above three new infections per one hundred persons-years at risk (Bärnighausen *et al.* 2008b, 2009). The HIV surveillance is nested within the Africa Centre Demographic Information System (ACDIS) (Tanser *et al.* 2008). The surveillance takes place annually in all consenting resident individuals aged 15 years or older. After offering an HIV test, fieldworkers elicit written informed consent from those eligible participants who agree to participate in the surveillance. They then obtain blood by finger prick and prepare dried blood spots for HIV testing according to 2001 UNAIDS/WHO Guidelines for using HIV testing technologies in surveillance (UNAIDS/WHO 2001).

### HIV treatment and care programme

The South African Department of Health started to provide HIV treatment and care in August 2004, supported by the Africa Centre with funding from the Presidential Emergency Plan for AIDS Relief (PEPFAR). The programme started at the Hlabisa referral hospital and was subsequently rolled out to all 17 primary care clinics in the sub-district (Houlihan *et al.* 2011). Following the national South African Department of Health guidelines, all adults with either stage-IV HIV disease (according to the WHO (2005) clinical HIV disease staging) or a CD4 count ≤200 cells/μl are offered ART (Department of Health South Africa 2010). In addition, since 2010 all patients with CD4 counts ≤350 cells/μl are eligible for ART, if they are either pregnant women or suffer from symptomatic tuberculosis (Department of Health South Africa 2010). Patients who are not yet eligible for ART initiation are enrolled in a pre-ART programme and monitored semi-annually. By January 2010, more than 13 500 patients were receiving ART through the programme. The demographic surveillance area (DSA) is about 40% of the programme catchment area, in terms of both people and geographical area (Bor *et al.* 2011). An estimated 21% of all HIV-infected individuals living in the Africa Centre DSA were receiving ART in 2008 (Cooke *et al.* 2010). With increasing ART coverage, HIV-related mortality in the community has significantly declined (Herbst *et al.* 2009, 2011).

Data in the HIV treatment and care programme were linked with demographic surveillance data using matching based on either the unique South African identification number or a patient’s first name, surname, age and sex. With such strict requirements for matching, the probability that a patient was mistakenly identified as a DSA resident is likely negligibly small. However, some significant proportion of patients who resided in the DSA may not have been matched because of data entry errors or use of different names in different settings. A previous analysis found that 26% of patients who reported living within the DSA could not be matched to the demographic surveillance (Cooke *et al.* 2010). Since this analysis, Africa Centre data management staff have identified additional programme patients within the surveillance (Bor *et al.* 2011), reducing the extent of misclassification of patients in this study as belonging to the group of HIV-infected people, in which some persons do and some do not know their HIV status, rather than to the group, in which all persons know their status.

### Sample and variables

Our sample comprised of 25 940 persons eligible for participation in the HIV surveillance, who met the following criteria. First, they were successfully contacted by the HIV surveillance fieldworkers in one round of the HIV surveillance. Second, they had either participated in the HIV surveillance during at least one previous surveillance round or they had enrolled in pre-ART care or been initiated on ART in the treatment and care programme.

Our outcome is an indicator variable for consent to an HIV test in the HIV surveillance during an eligible person’s most recent fieldworker visit during the period 2005–2010. Our main explanatory variables of interest include HIV status before the most recent fieldworker visit and indicator variables for enrolment in pre-ART care and ART initiation. We classified anyone who had a CD4 count or had been initiated on ART before the most recent fieldworker visit as HIV-infected, independent of past participation and HIV status data in the HIV surveillance. In all of our analyses, we controlled for sex and age (in 5-year age groups) at the time of the most recent fieldworker visit, because these demographic variables have been consistently found to strongly predict consent to HIV surveillance participation (Bärnighausen *et al.* 2008a). In addition, we controlled for the year of the most recent visit to account for secular trends in HIV surveillance behaviour.

### Analysis

We did three regressions using the same sample of 25 940 persons (which is described above). We first regressed consent to participation in the HIV surveillance during the most recent fieldworker visit on past HIV status, controlling for sex, age and year of the visit. Next, we stratified the persons in our sample into four groups: (i) HIV-uninfected; (ii) HIV-infected and neither enrolled in pre-ART care nor receiving ART; (iii) HIV-infected and enrolled in pre-ART care (as indicated by a previous CD4 count in the programme) but not receiving ART; and (iv) HIV-infected persons receiving ART (as indicated by an ART initiation date). We then regressed consent to participation on dummy variables capturing these four groups, again controlling for sex, age and year of the visit. Finally, we repeated the preceding regression, after additionally stratifying the groups enrolled in pre-ART and receiving ART into those whose last CD4 count in the HIV treatment and care programme was ≤200/μl (i.e. the ART eligibility threshold at the time) and those whose last CD4 count was >200/μl (i.e. indicating either that they were not yet eligible for ART, if they were enrolled in pre-ART, or that their ART was successful, if they were receiving ART).To determine whether the relationships between HIV status and participation in the HIV surveillance differed between women and men, we stratified all of the above regressions by sex.

## Results

**Figure 1:**
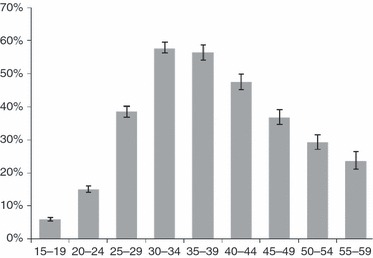
HIV prevalence by five-year age group in the sample of 25 950 persons who previously participated in the HIV surveillance. Error bars indicate 95% confidence intervals.

Table 1 shows summary statistics of our variables. About half of the eligible persons consented to participate in the HIV surveillance during the most recent fieldworker visit. Figure 1 shows the HIV prevalence by 5-year age group in the sample of 25 940 persons who had previously participated in the HIV surveillance. We find that HIV-infected individuals were significantly less likely to consent to participate in the surveillance than HIV-uninfected individuals [adjusted odds ratio (aOR) 0.74, *P* < 0.001], controlling for sex, age and year of the most recent fieldworker visit (Table 2, model 1). We further find that those persons who were receiving ART were less likely to consent to participate (aOR 0.75, *P* < 0.001) than those who had never sought treatment or care in the programme (aOR 0.82, *P* < 0.001), but more likely to consent than persons enrolled in pre-ART care (aOR 0.62, *P* < 0.001), see Table 2, model 2. Furthermore, those with CD4 count ≤200/μl were significantly less likely to consent to participate than those with CD4 count >200/μl in both the group enrolled in pre-ART (aOR 0.54, *P* < 0.001 *vs.* aOR 0.65, *P* < 0.001) and the group receiving ART (aOR 0.64, *P* < 0.001 *vs.* aOR 0.79, *P* < 0.001). See Table 2, model 3, for these results. We observe the same ranking of aORs among the different groups of HIV-infected persons in both women and men. In particular, when we run the regression in model 2 stratified by sex, we find that the aOR in the group who were receiving ART (0.74, *P* < 0.001) is lower than the aOR in the group who never sought treatment or care in the programme (0.82, *P* < 0.001) but higher than in the group enrolled in pre-ART care (0.62, *P* < 0.001). Similarly, in men, the aOR across the same three groups exhibited the same ranking (0.78 *vs.* 0.81 *vs.* 0.63, all *P* ≤ 0.013). When we run the regression model 3 stratified by sex, the ranking between those with CD4 count ≤200/μl and those with CD4 count >200/μl was maintained in all groups in both the regression on the sample of women and the regression on the sample of men.

**Table 1:** Description of variables

	% *N* = 25 940
Consent to an HIV test during most recent fieldworker visit	51
HIV-uninfected	73
HIV-infected	27
Neither enrolled in pre-ART nor receiving ART	12
Enrolled in pre-ART	7
CD4 ≤200	1
CD4 >200	6
Receiving ART	8
CD4 ≤200	2
CD4 >200	6
Women	62
Age	
15–19	26
20–24	24
25–29	13
30–34	8
35–39	7
40–44	6
45–49	6
50–54	6
55–59	4
Year of most recent fieldworker visit	
2005	3
2006	5
2007	6
2008	12
2009	26
2010	49

ART, antiretroviral treatment.

**Table 2:** Determinants of consent to participation in the HIV surveillance

	(1)		(2)		(3)	
			
	aOR	95% CI	aOR	95% CI	aOR	95% CI
HIV-uninfected	1		1		1	
HIV-infected	0.74	0.70–0.79***				
Neither enrolled in pre-ART nor receiving ART			0.82	0.75–0.89***	0.82	0.75–0.89***
Enrolled in pre-Art			0.62	0.56–0.69***		
CD4 ≤200/μl					0.54	0.43–0.67***
CD4 >200/μl					0.65	0.58–0.73***
Receiving ART			0.75	0.68–0.84***		
CD4 ≤200/μl					0.64	0.52–0.79***
CD4 >200/μl					0.79	0.70–0.88***
Women	1.48	1.40–1.56***	1.49	1.41–1.57***	1.49	1.41–1.57***
Age						
15–19	1		1		1	
20–24	0.78	0.73–0.84***	0.78	0.72–0.84***	0.78	0.73–0.84***
25–29	0.74	0.68–0.81***	0.74	0.68–0.81***	0.74	0.68–0.81***
30–34	0.70	0.63–0.78***	0.71	0.63–0.79***	0.71	0.63–0.79***
35–39	0.78	0.70–0.88***	0.78	0.70–0.88***	0.78	0.70–0.88***
40–44	0.69	0.61–0.77***	0.69	0.61–0.77***	0.69	0.61–0.77***
45–49	0.77	0.68–0.86***	0.77	0.68–0.86***	0.77	0.68–0.86***
50–54	0.83	0.74–0.94**	0.83	0.74–0.93**	0.83	0.74–0.93**
55–59	1.00	0.87–1.16	1.00	0.87–1.15	1.00	0.87–1.15
Year of most recent fieldworker visit						
2005	1		1		1	
2006	0.95	0.80–1.13	0.95	0.80–1.13	0.95	0.80–1.13
2007	0.62	0.53–0.74***	0.63	0.53–0.75***	0.64	0.54–0.75***
2008	0.59	0.51–0.69***	0.60	0.52–0.70***	0.60	0.52–0.70***
2009	0.49	0.42–0.56***	0.50	0.43–0.58***	0.50	0.43–0.58***
2010	1.5	1.27–1.67***	1.50	1.30–1.72***	1.50	1.30–1.72***

*N* = 25 940.

aOR, adjusted odds ratio; CI, confidence interval.

** *P* < 0.01;

*** *P* < 0.001.

We repeated the regressions shown in Table 2 with a different sample, which included, in addition to the sample of 25 940 persons described above, 13 981 persons, who had previously been eligible to participate in the HIV surveillance, but had never participated or enrolled in the treatment programme. This group was least likely to consent to participate during the most recent fieldworker visit (aOR, 1.94, *P* < 0.001, in all regressions). All of the odds ratios of the different HIV status, ART status and CD4-count groups were slightly larger in these regressions, but the difference in odds-ratio size never exceeded 5% and the rank order by size remained unchanged.

In the group who had never previously participated in the surveillance or enrolled in the ART programme, those who did participate during the most recent fieldworker visit (i.e. who participated for the first time during this visit) and had a valid HIV test result, overall 22% were HIV-infected (95% CI 20–23%). In those with a valid HIV test result, 43% fell into the youngest age group of persons 15–19 years of age (with an HIV prevalence of 5%, 95% CI 1–7%) and 57% were 20 years or older (with an HIV prevalence of 41%, 95% CI 38–44%).

## Discussion

We find that HIV-infected persons were significantly less likely than HIV-uninfected persons to consent to participation in a population-based HIV surveillance in a poor, rural community in KwaZulu-Natal, South Africa. This finding conforms with the hypothesis that HIV-infected persons are less likely to consent to participate in an HIV surveillance than HIV-uninfected persons because they fear the negative consequences of others learning their status. We further find that those among the HIV-infected who were either enrolled in pre-ART care or were already receiving ART were less likely to consent to participate than those who have never sought HIV treatment or care in the local programme. The group enrolled in pre-ART or ART know their status with certainty because CD4 counts are always preceded by HIV testing and provision of the test results, and ART is only initiated in persons who are aware of their status. The group who never sought HIV treatment or care, on the other hand, likely consists of persons who differ in their HIV status knowledge. Some people in this group may know with certainty that they are HIV-infected (because they have in the past accessed HIV testing and counselling), while others may suspect their status (based on evaluation of past risk behaviour or observation of HIV-related symptoms) and yet others may be completely ignorant of their infection. Thus, the fact that this latter group of people is more likely to consent to participate in the HIV surveillance than the other two groups of HIV-infected persons accords with our expectations, based on the hypothesis that an effect of HIV status on HIV surveillance participation is transmitted by HIV status knowledge.

Of course, we cannot rule out that alternative reasons that are inconsistent with our hypothesis explain these findings. Factors that are not sufficiently captured by sex, age and surveillance period could have confounded the relationships between HIV surveillance participation, HIV status and ART status. For instance, high levels of self-efficacy could lead persons to reject offers to participate in HIV surveillance, because it implies outside intervention in their lives and at the same time lead them to seek treatment in the HIV programme. It is also possible that sources of stigma associated with ART utilisation could reduce participation in HIV surveys and surveillances (Roura *et al.* 2009a). Future studies need to further investigate whether the relationships between HIV surveillance participation and HIV status is causal or not, for instance, by employing quasi-experimental approaches, or by eliciting reasons for HIV surveillance non-participation in in-depth interviews.

The finding that among the group of HIV-infected persons, who accessed the local HIV care and treatment programme, those who had not yet initiated ART were significantly less likely to consent to participate in the HIV surveillance than those who were already receiving ART is also in accordance with our hypothesis that HIV-infected persons are less likely to participate in HIV surveys and surveillances because they fear that others might learn their status. The reason for this conclusion is that ART is likely to lead to increased HIV status disclosure. Patients initiating ART in South Africa are required to disclose their HIV and ART status to at least one other person, a treatment supporter whose function is to help the ART patient to remain in care and to adhere well to treatment. Furthermore, over time, ART is likely to lead to disclosure to other family and community members. For instance, ART patients might decide to share their experience regaining good health on ART with other persons they suspect to be HIV-infected. Once a patient has widely disclosed that she is HIV-infected and takes ART against the disease, the fear that others might learn her status may no longer be a relevant motive for refusing participation in HIV surveys or surveillances. Additionally, the group of HIV-infected individuals who are enrolled in pre-ART care may include those who are particularly afraid that others might learn their HIV status. This fear might lead to both failure to initiate ART (which requires monthly instead of semi-annually clinic visits and is thus more difficult to conceal from family and community members) and failure to consent to participation in the HIV surveillance. Our finding that in the pre-ART group those with CD4 count ≤200/μl are significantly less likely to consent to participate in HIV surveillance than those with CD4 count >200/μl corresponds with this explanation, because the latter group has not progressed to receiving ART despite the diagnosis of ART eligibility.

While our findings are thus is in accordance with the hypothesised effect of HIV status on HIV surveillance participation, other mechanisms could also explain the results. For instance, the positive experience of regaining good health on ART may have improved attitudes towards participating in health research (Roura *et al.* 2009b), in general, or in the Africa Centre HIV surveillance, in particular, because the Africa Centre is visibly involved in the local HIV treatment and care programme providing doctors, nurses, ART counsellors and managerial support. Indeed, our finding that in the group receiving ART those whose treatment has been successful (as indicated by immunologic recovery with CD4 count >200/μl) are more likely to participate in HIV surveillance than those who fail treatment supports this hypothesis.

We demonstrate robustness of our findings to expansion of the regression sample to all those who had ever been eligible to participate in the HIV surveillance before their last contact with the surveillance fieldworker team, rather than only those who had previously provided blood for an HIV test or had been enrolled in the treatment programme. The group of those who consented to participate for the first time at the last fieldworker visit had a slightly lower HIV prevalence compared to the overall prevalence in those who had previously consented to participate. This finding can be explained by the fact that the proportion of young persons – who have a comparatively low prevalence – is much higher in this group than in the previous participants.

Overall, our findings provide further evidence for the past finding that HIV-infected persons are less likely to participate in HIV surveys and surveillance. However, the effect size in our study, while substantial, is smaller than the sizes observed in previous studies (Reniers & Eaton 2009; Bärnighausen *et al.* 2011) and does not differ by sex (Bärnighausen *et al.* 2011). For the first time, we elucidate one possible set of underlying reasons for the relationship between HIV status and participation in HIV surveillance with the use of data from an HIV treatment and care programme that is linked to data from a population-based surveillance, finding support for the hypotheses that HIV-infected people are less likely to participate in surveillance because they fear that others might learn their status.

Independent of whether this particular reason holds true or not, if utilisation of HIV treatment and care leads to reduced participation in HIV surveys and surveillance, HIV prevalence will be increasingly underestimated in countries where ART coverage is expanding, leading to biases in other HIV indicators whose estimation requires HIV prevalence values (such as ART coverage, when direct measures of ART need are not available, or HIV incidence, when it is derived in models based on changes in HIV prevalence over time). This problem is likely to become more relevant if ART eligibility is broadened, following the 2010 WHO recommendation (WHO 2010), or if treatment of all HIV-infected persons is pursued with the aim to reduce HIV transmission (Granich *et al.* 2010). Future studies need to assess the direction and sizes of biases in HIV indicators resulting from differential HIV survey and surveillance participation by HIV status. Even if utilisation of HIV treatment and care does not cause reduced participation in HIV surveys or surveillance, our findings are relevant for health policy. The fact that HIV-infected people who utilise HIV treatment or care are less likely than others to participate in HIV surveillance suggests that interventions motivating pre-ART and ART patients to participate in HIV-related research – delivered, for instance, as part of ‘positive prevention’ in pre-ART or ART patients (Bunnell *et al.* 2006) – could improve consent and reduce bias in HIV prevalence estimation.
